# Merging Heat Stress Tolerance and Health-Promoting Properties: The Effects of Exogenous Arginine in Cauliflower (*Brassica oleracea* var. *botrytis* L.)

**DOI:** 10.3390/foods10010030

**Published:** 2020-12-24

**Authors:** Jacinta Collado-González, María Carmen Piñero, Ginés Otálora, Josefa López-Marín, Francisco M. del Amor

**Affiliations:** Department of Crop Production and Agri-Technology, Murcia Institute of Agri-Food Research and Development (IMIDA), C/Mayor s/n, 30150 Murcia, Spain; mariac.pinero2@carm.es (M.C.P.); gines.oralora@carm.es (G.O.); josefa.lopez38@carm.es (J.L.-M.); franciscom.delamor@carm.es (F.M.d.A.)

**Keywords:** polyamines, *Brassica oleracea* var. *botrytis* L., short-term heat stress, UHPLC-DAD, exogenous arginine, antioxidants

## Abstract

In the last decades, cauliflower consumption has increased due to its observed beneficial effects on human health, especially on chronic diseases. Furthermore, the use of arginine has been shown to improve the heat stress tolerance of plants by increasing the polyamine content. Thus, we aimed to investigate the effects of the exogenous application of arginine on the physical and chemical quality parameters of cauliflower florets under heat stress. For this, we applied two concentrations of arginine (1 and 4 mM) to the leaves of cauliflower (*Brassica oleracea* var. *botrytis* L.) plants grown in three different temperatures (ambient, elevated, and extreme). Our data show that potassium and phosphate, as well as iron were the most abundant macro- and micronutrients, respectively. The combination of high temperature and exogenous application of arginine increased the antioxidant activity, total content of phenolic compounds, polyamines, and proteins. The data presented herein indicate that the combination of an adequate heat stress and the appropriate foliar arginine treatment may be a useful strategy that could be used to increase the number of valuable plant compounds in our diet.

## 1. Introduction

Cauliflower (*Brassica oleracea* var. *botrytis* L.) is a very important vegetable crop that belongs to the Brassica family [1]. This family is also named Cruciferae, and it is comprised of several crop species with important nutritional benefits for the human diet [2]. In recent years, a growing interest in adding cauliflower to the diet has been observed, due to the fact that this vegetable has a very complete nutritional profile; in particular, it can be considered as a good source of many valuable compounds that play an important role against chronic diseases, such as some types of cancer [1].

High temperature is one of the most important abiotic stresses that affect the cultivation of many plant species [3]. It induces a set of physiological and biochemical reactions in plants, with unfavorable effects such as oxidative stress. Due to the current global warming phenomenon, an increase in global temperature is expected [3]. Therefore, it is important to explore in-depth how heat stress can affect plant production and quality in order to find new strategies associated with adaptation to the climate change scenario.

Several authors have reported that antioxidant compounds are important in plants, as these can make them more tolerant to various abiotic stresses [3,4]. Specifically, arginine is an amino acid with a wide range of functions in living cells [4]. Thus, it not only serves as a protein constituent but is also a precursor of polyamines, agmatine, proline, and the cell signaling molecules glutamine and nitric oxide [5]. Polyamines are unsaturated hydrocarbons, with two or more primary amino groups [6]. Among the polyamines, putrescine, spermidine, and spermine, in particular, have been proposed as a new category of plant growth regulators. It has been suggested that they modulate various biological processes in plants, including growth, development, and apoptosis [6]. Moreover, polyamines are effective in the cellular defense against oxidative damage through the inhibition of lipid peroxidation and the elimination of free radicals [4].

The exogenous application of polyamines, or their precursor arginine, to plants has been shown to confer some protective effects against heat stresses [7]. However, the mechanism involved is not yet clear. To date, no data are available on the role of exogenous arginine as a precursor of these compounds in the antioxidative responses of cauliflower plants to extreme-temperature stress. To rectify this, the aim of this work was to study the effects of an arginine pretreatment on the alleviation of the stress generated at different temperatures. For this, two concentrations of arginine were studied (1 and 4 mM). Additionally, the role of arginine in combating oxidative stress was studied by monitoring the effects of its exogenous application on the content of important bioactive compounds.

## 2. Materials and Methods

### 2.1. Plant Materials, Experimental Conditions, and Treatments

Cauliflower plants, cv. Moonshine (El Jimenado S.A.), were grown in plastic containers previously filled with coconut coir fiber (Pelemix, Alhama de Murcia, Murcia, Spain) in a greenhouse composed of three equal modules. This greenhouse was located in Murcia, Spain (37°56′27.3″ N, 1°08′01.8″ W). The space between bags within a row was 33 cm, and the space between rows was 1 m. The cauliflowers were grown for 92 days in controlled conditions similar to those observed in the Mediterranean area. In this sense, these conditions consisted of a day/night temperature regime of 28/15 °C and relative humidity of 70%. Irrigation was supplied by self-compensating drippers (2 L h^−1^), and fresh modified Hoagland nutrient solution was applied to avoid salt accumulation, with a minimum of 35% drainage [8]. Specifically, the modified Hoagland solution had the following composition, in mM: NO_3_^−^: 12.0; H_2_PO^−^: 1.0; SO_4_^2−^: 3.5; K^+^: 7.0; Ca^2+^: 4.5; Mg^2+^: 2.0. Six days before the end of the assay, the plants were randomly divided into three groups, one of which (the control group) received foliar spraying with 20 mL of water. The plants from the other two groups were sprayed with 20 mL of 1 or 4 mM arginine. All the solutions applied contained 0.01% Tween-20 as a surfactant. After three days of the exogenous application of arginine, short-term heat stress was applied. The temperature of each module was as follows: module 1 was the control (ambient temperature, 26.9 °C, T_A_); in module 2, a gradient of ambient +5 °C was applied, obtaining a high temperature (T_H_, high temperature); and plants from module 3 were subjected to a gradient of ambient +8 °C (T_E_, extreme temperature). On the last day of the experiment, the samples were harvested at random and cauliflower florets were stored at −80 °C until analysis.

### 2.2. Chemicals and Reagents

Sodium hydroxide was purchased from Fluka (Buchs, Switzerland) and 2,2-azino-bis(3-ethylbenzothiazoline-6-sulfonic acid) diammonium salt (ABTS^•+^), 6-hydroxy-2,5,7,8-tetramethylchroman-2-carboxylic acid (Trolox), gallic acid, L-arginine, spermidine, spermine, cadaverine, histamine, putrescine, 1,6-hexaendiamine, benzoylchloride, glucose, sucrose, fructose, and inositol were obtained from Sigma-Aldrich (Steinheim, Germany). The SPE cartridges (C18 Sep-Pak cartridges) were bought from Waters Associates (Milford, MA, USA) and sodium carbonate, Folin–Ciocalteu reagent, ethyl ether, and LC-MS-grade methanol and acetonitrile were acquired from Panreac Química (Barcelona, Spain). Ultrapure water was produced using a Millipore water purification system.

### 2.3. Color Measurement

Cauliflower floret color was determined by taking three measurements. Surface color was measured as reflected color in the CIELAB (L* a* b*) color space, using a Konica Minolta CM-2600d sensing spectrophotometer (Minolta, Osaka, Japan). The results were expressed in the CIELAB system, and the mean values of the lightness (CIE L*), red/greenness (CIE a*), and blue/yellowness (CIE b*) parameters for each floret were calculated. The objective color was calculated as the chromaticity or Chroma (C*= (a*^2^ + b*^2^) ^1/2^), and the hue angle (H^◦^ = tan^−1^(b*/a*)).

### 2.4. Mineral Nutrient Content

The mineral content from cauliflower florets was measured from ground lyophilized material after the extraction of the minerals. For the determination of cations, an acid digestion with 0.1 g of samples was performed using an ETHOS ONE microwave digestion system (Milestone Inc., Shelton, CT, USA) followed by inductively coupled plasma (ICP) spectrometric (Varian Vista MPX, Palo Alto, CA, USA) analysis. The extraction of anions was performed with bidistilled water, and these were analyzed by using an ion chromatograph (METROHM 861 Advanced Compact IC; METROHM 838 Advanced Sampler).

### 2.5. Total Protein

The total protein content was measured in freeze-dried florets (after at least 72 h at 65 °C) using a combustion nitrogen/protein determinator (LECO FP-528, Leco Corporation, St. Joseph, MI, USA).

### 2.6. Total Soluble Sugars

The extraction of free soluble sugars present in cauliflower florets was carried out following the procedure described previously by Balibrea et al. [9], with some modifications. Thus, lyophilized cauliflower florets (50 mg) were extracted twice at 4 °C in 1.5 mL of 80% methanol, for 30 min each time, and with the mixture shaken every 10 min. Separately, each mixture was centrifuged for 15 min at 3500× *g* at 4 °C, and the resulting supernatant was passed through a C18 Sep-Pak cartridge (Waters Associates, Milford, Mass.), which was previously activated with methanol/water (80%/20%). Later, the filtrates were combined, filtered with an 0.45 μm filter (Millipore, Beford, MA, USA), and analyzed (20 μL) by ion chromatography (METRHOM 861 Advanced Compact IC; METROHM 838 Advanced Sampler). Total and individual free soluble sugars were expressed as grams per kilogram of dry weight.

### 2.7. Antioxidant Activity (ABTS^+^) and Total Phenols

Total phenolic compounds (TPC) were estimated according to Kähkönen et al. [10], using the Folin–Ciocalteu reagent. Samples (0.5 g) of cauliflower florets were homogenized with 5 mL of 80% acetone at room temperature and centrifuged at 10,000× *g* at 4 °C for 10 min. The supernatant (100 μL) was mixed with 1 mL of Folin–Ciocalteu reagent diluted with Milli-Q water (1:10) and 2 mL of Milli-Q water. This mixture was left to react at room temperature for 3 min, and then 2 mL of 20% sodium carbonate was added and mixed thoroughly. The mixture was incubated at room temperature again, but this incubation was in the dark and for 30 min. The absorbance of the resulting blue-colored solution was measured at 765 nm using a UV–Vis spectrophotometer (Shimadzu UV-1800 model with the CPS-240 cell holder, Shimadzu Europa GmbH, Duisburg, Germany). The phenolic compounds were quantified on the basis of the standard curve for gallic acid. The results are expressed as mg of gallic acid equivalents (GAE) g^−1^ FW.

The ABTS^+^ (2,2-azinobis-(3-ethylbenzothiazoline-6-sulfonic acid)) radical cation assay was carried out following the method previously described by Cano-Lamadrid et al. [11]. Freeze-dried samples (0.5 g) were homogenized with 10 mL of 80% methanol +1% HCl at room temperature, sonicated for 15 min at 20 °C, and kept for 24 h at 4 °C. Then, this mixture was sonicated for 15 min and centrifuged for 10 min at 10,000× *g*. The stock solution of ABTS^+^ was diluted with water to reach an absorbance of 0.7 at 734 nm. Then, 10 μL of each supernatant was mixed with 990 μL of the ABTS^+^ solution; after 10 min of incubation under dark conditions, the absorbance was recorded at 734 nm using a UV–Vis spectrophotometer (Shimadzu CPS-240 model, Japan). The results were standardized to Trolox equivalents per gram of dry weight.

### 2.8. Polyamine Analysis

The extraction of polyamines was performed as reported by Rodríguez et al. [12], with only minor modifications. For each replicate, fresh samples were milled, and 5 g was extracted with 7.5 mL of cold perchloric acid (5%, *v/v*), using an ultraturrax (Ika, Staufen, Germany), for 1 min. The homogenate was then centrifuged (Eppendorf centrifuge 5804R, Hamburg, Germany) for 8 min at 12,000× *g*. A 500 µL aliquot of the supernatant was used to determine free polyamines by benzoylation. For this reaction, 20 µL of an internal standard (1,6-hexanediamine), 2 mL of 2 N NaOH, and 20 µL of benzoyl chloride were added to that supernatant, and the solution was vortexed for 15 s and incubated for 20 min at room temperature. Later, in order to finish the benzoylation reaction, this mixture was mixed with 4 mL of saturated NaCl solution. The polyamines present in cauliflower florets were obtained by adding 2 mL of cold diethyl ether, and both phases (aqueous and organic) were saved at −20 °C until the total extraction of the polyamines in the ether phase. Lastly, the ether phase (1.5 mL) was evaporated to dryness, using a SpeedVac concentrator (Savant SPD121P, Thermo Scientific, Waltham, MA, USA), and redissolved in 500 µL of water/acetonitrile (58%/42%, *v/v*). The polyamine derivatives were analyzed by UHPLC according to Rodríguez et al. [12], with slight modifications. The injection volume was 10 μL. To obtain a satisfactory separation, water/acetonitrile (58%/42%, *v/v*) solvents running isocratically with a flow rate of 0.55 mL min^−1^ were utilized. The benzoyl-polyamines were eluted through a reversed-phase ACQUITY UPLC HSS T3 column (2.1 × 100 mm, 1.8 µm) (Waters Corp., Wexford, Ireland). The column was maintained at 40 °C, and the compounds were analyzed by using a UHPLC-DAD (Waters Technologies, Waldbronn, Germany); the absorbance was measured at 254 nm. Data acquisition and processing were carried out by using Empower 2 (Waters) software.

### 2.9. Statistical Analysis

The experimental design was random and all analyses were performed with five replicates. An analysis of variance (ANOVA) was performed with the statistical software SPSS [13], followed by a Tukey’s multiple-range test to compare the means and determine significant differences with regard to all factors. Differences were considered statistically significant at *p* < 0.05.

## 3. Results and Discussion

### 3.1. Color Parameters

Our data show how cauliflower floret color was affected by the heat and arginine treatments. The effect of heat on the color parameters can be observed in Table 1. When arginine (Arg) was not applied, lightness (L*) by 17% in high (T_H_) or extreme (T_E_) temperatures, and chroma (C°) in T_E_ by 27%_._ However, a* and hue (H°) decreased in T_H_, although these recovered in T_E_.

These results are similar to those from Hodges et al. [14], who reported that the curd color of the Indian cauliflower cv. Fremont was yellowish to creamy white. Additionally, Singh et al. [15] indicated that cauliflower showed a wide adaptability to temperature and humidity. Regarding the application of Arg, the data show that the foliar application did not produce significant differences in any parameter, except for L*. These results are similar to the results found by other authors. Taking into account that Singh et al. [15] reported that mature cauliflowers were yellowish and had a greater luminosity, our results indicate that preharvest spraying of arginine may retard color changes. White-colored cauliflowers are favored by customers, and also, for a cauliflower to be well considered by consumers, it must have a compact, white-colored, and medium-sized curd and be free from any disease or disorder [16]. Moreover, although the white cauliflower has low levels of colored pigments (e.g., carotenoids, chlorophylls), it is widely consumed due to its content of certain flavonoids and glucosinolates, making it a vegetable that is very rich in antioxidant activity [17].

### 3.2. Mineral Content

In this study, 10 cations belonging to two classes—macro- and micronutrients—and 5 anions were studied (Table 2, Table 3 and Table 4). The total macroelement cations varied from 46.01 to 54.65 g kg^−1^ DW, and the individual macroelement content ranged between 1.71 and 41.70 g kg^−1^ DW. The most abundant cation was potassium (K) (Table 2). Calcium (Ca) was the only macroelement that was affected by heat stress. Thus, Ca content significantly increased as the temperature increased from T_A_ to T_H_ or T_E_ (Table 2). The preharvest spraying of arginine produced a decrease in sodium (Na) content, with the lowest value of Na obtained when arginine was applied at 4 mM (Table 2).

Table 3 shows the most abundant anions present in cauliflower florets. The total anion concentration varied from 9.0 to 30.1 g kg^−1^ DW, varying individually from 0.3 to 20.1 g kg^−1^ DW. The least abundant and predominant anions were nitrite and phosphates, respectively. In the absence of foliar arginine application, the florets from plants exposed to heat stress showed a significant reduction in the sulfates and phosphates and a sharp increase in chloride and nitrate (Table 3). With respect to the effect of the foliar spraying, Table 3 shows that the plants sprayed with arginine had a higher content of chloride, regardless of the temperature, and a high content of nitrate and phosphate at T_E_. Nitrite was only found in plants sprayed with 1 mM arginine, and at T_H_ and T_E_ (Table 3).

The total microelements varied in concentration from 83.85 to 115.70 mg kg^−1^ DW, with individual microelements oscillating from 0.50 to 35.83 mg kg^−1^ DW (Table 4). The most abundant were iron (Fe) and zinc (Zn), and the least abundant was copper. Heat stress affected all the microelements except for Fe. In this sense, boron (B), Zn, and copper (Cu) were reduced at T_H_ and T_E_, while manganese (Mn) decreased only at T_E_ (Table 4). No significant changes were observed in the total microelements as a result of foliar arginine application. Similar results were obtained for Mn and B, but other microelements were affected. In this sense, the concentration of Zn increased regardless of the concentration of arginine at T_H_ and T_E_, whereas the Cu and Fe content was reduced after arginine application (Table 4).

The contents of macro- and microelements obtained in the cauliflower cv. Moonshine were higher than those of the Australian cauliflowers studied by Cunningham et al. [18] and those reported by the USDA for raw cauliflower [19]. Nevertheless, our results are consistent with those previously reported for white cauliflower [20]. There is a small number of studies aimed at investigating the effect of heat stress on the mineral concentration of plants. Our results obtained due to heat stress application are in agreement with previous reports which indicate that heat stress alters the uptake of nutrients by plants. Among the nutrients whose accumulation was affected by heat stress, with an important decrease in their contents in plants, were potassium, sodium, and phosphorus. The uptake of nutrients could be influenced by several factors, which ultimately results in a decrease in the content of the nutrients in the plant as compared with a control treatment [21].

Regarding the results after spraying with arginine on the accumulation of nutrients in cauliflower florets, calcium, magnesium, phosphorous, and potassium tended to increase, although a significant reduction in sodium accumulation was also observed. These results matched previous studies carried out on *Rosa hybrid*, which indicate that heat-sensitive plants apparently have a reduced capacity of using these nutrients for physiological processes under heat stress [22]. The heat tolerance of these sensitive plants can be improved by the exogenous application of osmoprotectors such as arginine or polyamines [7]. A reduction in the Na/K ratio could also be observed in our results. This is supported by a study carried out on pistachio treated exogenously with free polyamines: Kamiab et al. [23] reported that this not only decreased Na^+^ accumulation but also Cl^−^ accumulation and the Na^+^/K^+^ ratio. Our data also show an increase in the accumulation of Cl^−^ and nitrate under moderate heat stress in plants sprayed with 1mM of arginine. A point worth mentioning is that arginine is not only an amino acid but also a precursor of polyamines and the cell signaling molecules glutamine and nitric oxide (NO) [5]. In accordance with this, in a study performed on mung bean under water stress, it was shown that both nitrogen uptake and nitrogen content improved in plants treated with polyamines [24].

From a health perspective, the nutrients that should be highlighted are iron, magnesium, and zinc. These cations are crucial for cognitive, behavioral, and motor development. In this sense, iron-deficit anemia and low zinc and magnesium contents are more common in children with Autism Spectrum Disorder [25,26]. Our results show an increase in the zinc content of cauliflower florets after the application of foliar arginine and an increase in magnesium after the application of both treatments: heat stress and arginine spraying.

### 3.3. Total Protein Content

**Figure 1:**
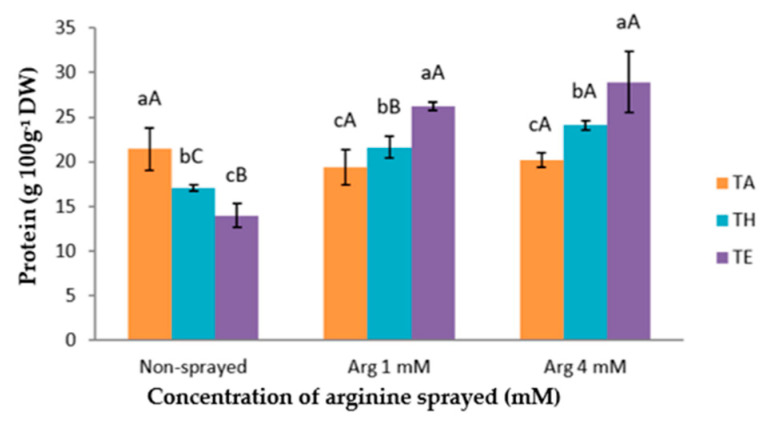
Content of protein (g 100 g^−1^ DW) in the florets of cauliflower plants subjected to different temperatures and sprayed with two different concentrations of foliar arginine (1 and 4 mM). The data are presented as the treatment means (*n* = 4). Different small letters indicate significant differences between temperatures, and different capital letters indicate significant differences between arginine treatments, at the same temperature. The mean values of the main effects are shown. Abbreviations: Arg: arginine, T: temperature.

The protein concentration ranged from 14.00 to 28.91 g 100 g^−1^ DW (Figure 1). The heat stress and both foliar arginine treatments affected N uptake and, consequently, the protein content in the cauliflower florets. In this sense, when only the heat stress was applied, in the absence of arginine, a decrease of the protein content was observed. This finding is in agreement with the results reported by several authors, who indicated that heat stress in plants leads to a significant decrease in the nitrogen content [27]. Additionally, the lower content of proteins in the plants subjected to heat stress could be mainly attributed to the fact that at high temperatures some proteins are not only not synthesized but also denatured [28]. This decrease in the protein content in plants will depend not only on the characteristics of the stress (intensity and duration) but also on the plant species, and mainly on the phenological period in which the heat stress was applied [6]. Moreover, our data surprisingly show the highest concentration of proteins after the exogenous application of 4 mM arginine in the cauliflower cv. Moonshine. This is consistent with the results obtained in previous works, where it was found that the protein content increased in wheat plants and mung beans after plants were treated with polyamines, improving their thermotolerance [24,29]. In a study carried out on sugarcane (*Saccharum* sp.) and alfalfa, it was observed that arginine may have played a positive role in the somatic embryogenesis of sugarcane [30]. At first, this finding was only expected for the 1 mM arginine treatment and not for the 4 mM one, because all the previous authors had used polyamines or arginine in a range between 1 and 2.5 mM, and 4 mM arginine seemed to be too high for obtaining good results.

### 3.4. Qualitative and Quantitative Profiles of Free Soluble Sugars

Four free sugars—inositol, glucose, fructose, and sucrose—were evaluated in this study, and glucose was the predominant one in cauliflower cv. Moonshine (Table 5). The total free sugar content varied within a range from 163.59 to 240.18 g kg^−1^ DW in the florets, showing an increase in those subjected to the T_E_ and foliar arginine at 4 mM (Table 5).

Hodges et al. [14] and Bhandari and Kwak [17] reported that lyophilized cauliflower florets contain over 300 mg g^−1^ of total free sugars, suggesting that free sugar levels in cauliflower are genotype dependent. Our data show a reduction in free total soluble sugars as a result of the application of the heat stress treatments. Several authors have reported that the production of osmolytes, including free sugars, in plants under heat stress could be related to the stability of invertase activity, as it stabilizes the structure of the membrane bilayer, exerting protective effects [31]. However, it is necessary to know that the regulation of starch metabolism induced by abiotic stress can favor starch biosynthesis. [32]. The fact that the free sugars increased by 25% or 47% depending if 1 or 4 mM of arginine was applied before the short-term heat stress can be attributed to the role of arginine as an osmoregulator [32,33]. Our results are similar to those found in a study carried out in wheat plants, where increases in sugars were obtained as a result of spraying different concentrations of arginine [29]. Although there are some works about exogenous application of polyamines and, specifically, arginine, the mechanism of action of polyamines is still unknown [32].

### 3.5. Total Phenolic Compounds and Antioxidant Activity (ABTS^+^)

It is known that *Brassica* crops are a very good source of bioactive substances [34,35,36]. The data on total phenolic compounds (TPC) and antioxidant activity of cauliflower florets ranged between 77.53 and 132.36 mg GAE g^−1^ FW, and between 38.17 and 138.73 µM g^−1^ DW, respectively (Table 6). However, as our work was designed and carried out to avoid any effects produced by drought, both the antioxidant activity and the TPC content were enhanced by heat stress (Table 6). Similar results were previously found for white cauliflower florets [17]. It has been reported that heat stress generates a significant increase in antioxidant enzyme activities, confirming that plants subjected to stressful temperatures suffer oxidative stress [37]. As shown in Table 6, plants treated with foliar arginine showed an increase in both the antioxidant activity and the TPC content in a concentration-dependent manner. Thus, a higher increase in antioxidant activity and the TPC content when 4 mM arginine was sprayed can be observed. These findings match those found by other authors with other plant materials (hot pepper fruits, rice (*Oryza sativa* L.), and Indian mustard (*Brassica juncea* L.)), who reported that spraying arginine or putrescine increased the TPC of plants under abiotic stress [6,38,39]. It is worth stating that putrescine can be synthesized from arginine via agmatine, through the action of arginine decarboxylase [40].

The importance of phenols lies in their cell protection role against the oxidative damage suffered by plants due to stress. Furthermore, these compounds are also capable of increasing the stability of the cell membrane [39]. These results suggest that moderate or extreme heat stress combined with the foliar application of 1 or 4 mM arginine can improve the nutritional value and the content of beneficial substances of cauliflower cv. Moonshine.

### 3.6. Determination of Polyamines

**Figure 2:**
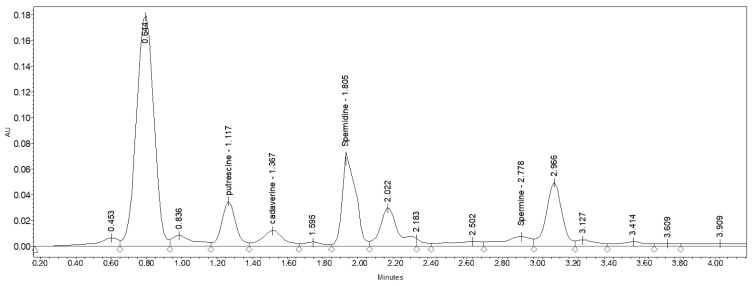
Chromatogram of polyamines found in cauliflower florets under short-term heat stress at extreme temperature and preharvest foliar 4 mM arginine treatment.

Table 7 and Figure 2 show the chromatographic profile of the polyamines present in cauliflower florets. The total polyamine concentration in the cauliflower florets oscillated between 21.14 and 259.05 nmol g^−1^ FW, with the individual contents ranging from 1.88 to 124.69 nmol g^−1^ FW. Putrescine was the most abundant polyamine, except at ambient temperature without a foliar treatment or when plants were sprayed with arginine at 1 mM. In these cases, cadaverine was the most abundant polyamine (Table 7). Specifically, the concentrations of putrescine, cadaverine, spermidine, and spermine varied from 3.19 to 124.69 nmol g^−1^ FW, 11.19 to 26.72 nmol g^−1^ FW, 1.88 to 80.89 nmol g^−1^ FW, and 4.88 to 26.75 nmol g^−1^ FW, respectively (Table 7). The polyamine content in cauliflower plants under heat stress was higher as the temperature increased. These results are in accordance with those obtained in several studies carried out with plant materials such as *Brassica alboglabra* Bailey leaves, *O. sativa*, *B. juncea*, *Vitis vinifera* L., *Valeriana officinalis* L., *Matricaria chamomilla* L., and *Origanum majorana* L., which reported that the concentrations of polyamines were usually higher under biotic stresses, including heat stress [41,42]. Additionally, our results also show that as a consequence of the foliar arginine treatments, the accumulation of endogenous polyamines increased, showing a higher accumulation when 4 mM arginine was applied (Table 7). The findings of this study are similar to those of other works performed on stressed plants (rice, chickpea, alfalfa, *Nymphoides pelsatum*, wheat, tomato, citrus, welsh onion, eggplant, fenugreek sprouts, and bean), where it was shown that the exogenous application of polyamines is an effective approach for endowing plants with tolerance against abiotic stresses such as salinity [33], cold [43], drought [44], heavy metals [45], osmotic stress [46], high temperatures [29], waterlogging [47], and flooding [48]. However, until now, there have been no reports focused on the exogenous preharvest application of arginine in cauliflower to reduce heat damage to the florets. Several authors have suggested that the increase in endogenous polyamines as a result of the foliar application of arginine in wheat plants could be due to a decline in ethylene biosynthesis and a synergistic interaction between ethylene and polyamines [29].

Our data have shown that cauliflower cv. Moonshine under short-term heat stress and sprayed with 4 mM arginine contained 339.60 nmoles g^−1^ of polyamines. This value is higher than those observed by Eliassen et al. [49]. These authors reported that fresh cauliflower contained around 290 nmoles g^−1^ of polyamines, indicating that broccoli and cauliflower showed a content of polyamines that was even higher than that obtained for meat or fish. There is emerging evidence that polyamines can be utilized as a therapeutic option for humans. In fact, an earlier work associated the intake of polyamines with a low incidence of cardiovascular events and reduced mortality [50]. Therefore, it has been estimated that a standard human diet should provide a daily dose of polyamines [51].

## 4. Conclusions

Our data have shown that the foliar application of arginine, regardless of its concentration (1 or 4 mM), before heat stress enhanced the accumulation of nutrients such as calcium, magnesium, phosphorous, potassium, and zinc, and reduced the accumulation of sodium, highlighting the osmoregulatory role of arginine. The reduction in the content of sugars and proteins in the absence of the foliar application may be mainly ascribed to an increase in starch biosynthesis, and protein denaturation and inhibition of protein synthesis, respectively. Nevertheless, the increasing concentrations of different sugar and protein contents were the result of the osmoregulatory role of arginine in plants. Our results also show an increase in antioxidant enzymatic activities, phenolic compounds, and endogenous polyamine content after the application of short-term heat stress and spraying of arginine, especially 4 mM arginine. With these results, it can be concluded that the exogenous application of arginine at 4 mM allows obtaining plants with a greater tolerance to heat stress. Therefore, the combination of the exogenous application of 4 mM arginine along with the application of short-term heat stress at extreme temperatures encourages the production of compounds that are beneficial for plant growth. Moreover, this strategy could also be taken into account to improve the quantity of beneficial compounds and their effects in our plant diet.

## Figures and Tables

**Table 1:** Color parameters (lightness (L*), red-greenness (a*), blue-yellowness (b*), chroma (C*), and hue angle (H^0^) values) of cauliflower florets as affected by short-term heat stress and foliar arginine application.

Treatment	Temp	L*	a*	b*	C*	H^0^
Control	T_A_	53.1 ± 8.2 ^bB^	1.0 ± 0.1 ^aA^	21.9 ± 2.6 ^aA^	22.8 ± 1.7 ^bA^	87.8 ±1.3 ^aA^
	T_H_	62.6 ± 1.9 ^aB^	−0.5 ± 0.4 ^bB^	22.7 ± 1.6 ^aA^	19.4 ± 2.5 ^bA^	−88.6 ± 0.9 ^bA^
	T_E_	62.3 ± 4.5 ^aB^	1.0 ± 0.7 ^aA^	24.2 ± 4.4 ^aA^	28.9 ± 4.5 ^aA^	87.0 ± 0.7 ^aA^
Arg 1 mM	T_A_	64.7 ± 2.1 ^aA^	−0.4 ± 0.3 ^bB^	20.3 ± 3.4 ^aA^	22.3 ± 3.3 ^aA^	87.1 ± 0.9 ^bA^
	T_H_	69.4 ± 4.6 ^aA^	0.7 ± 0.2 ^aA^	23.3 ± 1.8 ^aA^	23.3 ± 1.8 ^aA^	−88.9 ± 0.5 ^cA^
	T_E_	67.0 ± 4.7 ^aA^	1.1 ± 0.2 ^aA^	25.8 ± 4.9 ^aA^	24.2 ± 4.4 ^aA^	87.8 ± 1.3 ^aA^
Arg 4 mM	T_A_	70.7 ± 2.6 ^aA^	1.0 ± 0.2 ^aA^	22.3 ± 1.8 ^bA^	22.3 ± 4.0 ^aA^	87.4 ± 1.3 ^bA^
	T_H_	74.5 ± 3.7 ^aA^	−0.6 ± 0.2 ^bB^	22.4 ± 5.8 ^bA^	19.4 ± 4.0 ^aA^	−88.4 ± 0.6 ^cA^
	T_E_	70.5 ± 4.5 ^aA^	0.9 ± 0.1 ^aA^	31.7 ± 4.4 ^aA^	25.8 ± 4.9 ^aA^	88.2 ± 0.7 ^aA^
**Main effects**						
Temperature (T)		***	***	**	**	***
Arginine (Arg)		ns	ns	ns	ns	ns
T × Arg		***	***	ns	ns	ns

Different small letters within a column indicate significant differences between temperatures, and different capital letters within a column, for the same temperature, indicate significant differences between arginine treatments, at *p* = 0.05 (Tukey’s test). Analysis of variance: ns, not significant; ** *p* ≤ 0.005; *** *p* ≤ 0.001. Abbreviations: Arg (arginine). T_A_, T_H_, T_E_ (temperature ambient, high, and extreme).

**Table 2:** Content of macroelements (g kg^−1^ DW) of cauliflower florets as affected by short-term heat stress and foliar arginine application.

Treatment	Temp	Na	K	Ca	Mg	P	Total
Control	T_A_	2.7 ± 0.8 ^aA^	38.1 ± 2.6 ^aA^	1.7 ± 0.1 ^bA^	1.8 ± 0.2 ^aA^	6.2 ± 1.2 ^aA^	46.0 ± 4.7 ^aA^
	T_H_	2.6 ± 0.5 ^aA^	36.3 ± 4.3 ^aA^	2.3 ± 0.6 ^aA^	1.9 ± 0.3 ^aA^	5.7 ± 0.8 ^aA^	48.7 ± 6.2 ^aA^
	T_E_	2.7 ± 0.4 ^aA^	34.3 ± 2.0 ^aA^	2.4 ± 0.5 ^aA^	2.1 ± 0.3 ^aA^	5.5 ± 0.3 ^aA^	51.6 ± 3.3 ^aA^
Arg 1 mM	T_A_	2.5 ± 0.3 ^aB^	41.7 ± 4.9 ^aA^	2.1 ± 0.2 ^bA^	2.0 ± 0.2 ^aA^	6.3 ± 1.2 ^aA^	47.6 ± 6.5 ^aA^
	T_H_	2.4 ± 0.2 ^aB^	39.2 ± 4.7 ^aA^	2.3 ± 0.3 ^bA^	2.1 ± 0.2 ^aA^	6.2 ± 0.6 ^aA^	52.0 ± 5.7 ^aA^
	T_E_	2.2 ± 0.1 ^aB^	35.2 ± 4.9 ^aA^	2.8 ± 0.3 ^aA^	2.3 ± 0.3 ^aA^	5.8 ± 0.5 ^aA^	55.2 ± 5.8 ^aA^
Arg 4 mM	T_A_	2.3 ± 0.2 ^aB^	41.2 ± 3.5 ^aA^	2.6 ± 0.5 ^aA^	2.1 ± 0.3 ^aA^	6.4 ± 0.9 ^aA^	50.6 ± 5.1 ^aA^
	T_H_	2.1 ± 0.2 ^aB^	40.5 ± 3.5 ^aA^	2.8 ± 0.3 ^aA^	2.1 ± 0.2 ^aA^	6.3 ± 0.7 ^aA^	53.7 ± 4.07 ^aA^
	T_E_	2.0 ± 0.1 ^aB^	37.6 ± 5.4 ^aA^	2.8 ± 0.4 ^aA^	2.3 ± 0.3 ^aA^	6.0 ± 1.1 ^aA^	54.7 ± 6.9 ^aA^
**Main effects**							
Temperature (T)		**	ns	***	*	ns	ns
Arginine (Arg)		*	ns	ns	ns	ns	ns
T × Arg		**	ns	ns	ns	ns	ns

Different small letters within a column indicate significant differences between temperatures, and different capital letters within a column, for the same temperature, indicate significant differences between arginine treatments, at *p* = 0.05 (Tukey’s test). Analysis of variance: ns, not significant; * *p* ≤ 0.05; ** *p* ≤ 0.005; *** *p* ≤ 0.001. Abbreviations: Arg (arginine), Ca (calcium), K (potassium), Mg (magnesium), Na (sodium), p (phosphorus), T (temperature).

**Table 3:** Anion content (g kg^−1^ DW) of cauliflower florets affected by short-term heat stress and foliar arginine application.

Treatment	Temp	Chloride	Nitrate	Phosphates	Sulfates	Total
Control	T_A_	1.3 ± 0.3 ^bB^	0.3 ± 0.01 ^bA^	16.3 ± 5.1 ^aA^	7.5 ± 0.5 ^aA^	25.4 ± 5.6 ^aA^
	T_H_	1.4 ± 0.5 ^bB^	0.4 ± 0.10 ^bA^	15.7 ± 3.6 ^aA^	7.2 ± 0.9 ^aA^	24.7 ± 4.8 ^aB^
	T_E_	2.0 ± 0.2 ^aA^	1.1 ± 0.18 ^aB^	0.6 ± 0.1 ^bB^	5.3 ± 0.6 ^bA^	9.0 ± 1.0 ^bB^
Arg 1 mM	T_A_	1.9 ± 0.7 ^bA^	0.3 ± 0.06 ^bA^	16.2 ± 3.4 ^aA^	8.2 ± 1.4 ^aA^	26.6 ± 5.6 ^aA^
	T_H_	2.0 ± 0.2 ^bA^	0.5 ± 0.07 ^bA^	17.4 ± 1.2 ^aA^	8.0 ± 0.5 ^aA^	27.8 ± 2.0 ^aA^
	T_E_	2.0 ± 0.2 ^aA^	1.2 ± 0.23 ^aA^	20.1 ± 0.7 ^aA^	5.9 ± 0.7 ^bA^	29.5 ± 1.9 ^aA^
Arg 4 mM	T_A_	1.9 ± 0.5 ^aA^	0.3 ± 0.01 ^bA^	15.7 ± 2.5 ^bA^	8.1 ± 0.5 ^aA^	26.0 ± 3.3 ^bA^
	T_H_	2.1 ± 0.3 ^aA^	0.4 ± 0.01 ^bA^	18.5 ± 1.5 ^aA^	6.5 ± 0.4 ^bA^	27.5 ± 2.2 ^bA^
	T_E_	2.4 ± 0.3 ^aA^	1.5 ± 0.37 ^aA^	19.8 ±1.3 ^bA^	6.4 ± 0.5 ^bA^	30.1 ± 2.3 ^aA^
**Main effects**						
Temperature (T)		*	***	***	***	***
Arginine (Arg)		**	ns	***	ns	***
T × Arg		ns	*	***	*	***

Different small letters within a column indicate significant differences between temperatures, and different capital letters within a column, for the same temperature, indicate significant differences between arginine treatments, at *p* = 0.05 (Tukey’s test). Analysis of variance: ns, not significant; * *p* ≤ 0.05; ** *p* ≤ 0.005; *** *p* ≤ 0.001. Abbreviations: Arg (arginine), T (temperature).

**Table 4:** Content of microelements (mg kg^−1^ DW) of cauliflower florets affected by short-term heat stress and foliar arginine application.

Treatment	Temp	Fe	Cu	Mn	Zn	B	Total
Control	T_A_	35.8 ± 3.0 ^aA^	2.5 ± 1.3 ^aA^	20.9 ± 3.2 ^aA^	33.1 ± 3.5 ^aA^	23.4 ± 2.0 ^aA^	115.7 ± 12.4 ^aA^
	T_H_	33.2 ± 5.5 ^aA^	1.7 ± 0.2 ^bA^	12.8 ± 1.5 ^aA^	23.0 ±1.0 ^bB^	20.3 ± 1.5 ^bA^	90.9 ± 9.2 ^bA^
	T_E_	29.7 ± 3.9 ^aA^	1.6 ± 0.3 ^bA^	13.7 ± 2.5 ^bA^	23.7 ± 1.7 ^bB^	18.6 ± 0.6 ^bA^	87.3 ± 8.6 ^bA^
Arg 1 mM	T_A_	30.5 ± 10.0 ^aA^	1.6 ± 0.6 ^aB^	19.6 ± 0.9 ^aA^	36.1 ± 8.0 ^aA^	22.2 ± 2.0 ^aA^	110.0 ± 20.4 ^aA^
	T_H_	27.7 ± 3.5 ^aA^	1.6 ± 0.3 ^aA^	20.1 ± 3.2 ^aA^	32.6 ± 5.0 ^aA^	21.9 ± 2.9 ^aA^	103.9 ± 14.2 ^aA^
	T_E_	22.0 ± 0.5 ^aB^	1.4 ± 0.3 ^aA^	14.3 ± 1.9 ^bA^	27.3 ± 2.7 ^aA^	20.2 ± 1.7 ^aA^	85.1 ± 6.7 ^bA^
Arg 4 mM	T_A_	29.6 ± 8.6 ^aA^	1.3 ± 0.3 ^aB^	17.1 ± 1.2 ^aA^	31.3 ± 10.2 ^aA^	20.5 ± 2.5 ^aA^	99.7 ± 21.7 ^aA^
	T_H_	20.9 ± 3.3 ^aB^	0.5 ± 0.2 ^bB^	17.5 ± 3.0 ^aA^	29.9 ± 4.9 ^aA^	20.2 ± 1.2 ^aA^	89.0 ± 12.0 ^bA^
	T_E_	19.5 ± 2.7 ^aB^	0.5 ± 0.1 ^bB^	14.6 ± 2.9 ^bA^	28.5 ± 5.2 ^aA^	20.8 ± 2.9 ^aA^	83.9 ± 13.1 ^bA^
**Main effects**							
Temperature (T)		*	ns	***	*	ns	***
Arginine (Arg)		***	***	ns	ns	ns	ns
T × Arg		**	**	**	ns	ns	**

Different small letters within a column indicate significant differences between temperatures, and different capital letters within a column, for the same temperature, indicate significant differences between arginine treatments, at *p* = 0.05 (Tukey’s test). Analysis of variance: ns, not significant; * *p* ≤ 0.05; ** *p* ≤ 0.005; *** *p* ≤ 0.001. Abbreviations: Arg (arginine), B (boron), Cu (copper), Fe (iron), Mn (manganese), Zn (zinc), T (temperature).

**Table 5:** Mean concentrations of sugars (g kg^−1^ DW) of cauliflower florets affected by short-term heat stress and foliar arginine application.

Treatment	Temp	Inositol	Glucose	Fructose	Sucrose	Total Free Sugars
Control	T_A_	12.5 ± 0.8 ^aA^	101.1 ± 12.1 ^aA^	57.3 ± 3.0 ^aA^	51.7 ± 4.3 ^aA^	222.6 ± 19.8 ^aA^
	T_H_	11.9 ± 2.9 ^aB^	90.9 ± 6.0 ^aB^	49.4 ± 3.1 ^aA^	17.2 ± 1.5 ^bC^	169.4 ± 13.2 ^bC^
	T_E_	11.0 ± 2.1 ^aB^	98.3 ± 2.1 ^aB^	54.3 ± 5.1 ^aB^	nd	163.6 ± 9.1 ^bC^
Arg 1 mM	T_A_	7.8 ± 1.9 ^bB^	59.0 ± 0.6 ^cC^	32.2 ± 0.4 ^cC^	43.7 ± 3.0 ^aB^	142.8 ± 5.8 ^bB^
	T_H_	15.0 ± 1.2 ^aA^	101.0 ± 4.8 ^bA^	54.0 ± 3.6 ^bA^	25.8 ± 2.0 ^bB^	195.8 ± 11.4 ^aB^
	T_E_	15.4 ± 1.4 ^aA^	106.3 ± 3.3 ^aA^	73.1 ± 3.9 ^aA^	nd	194.8 ± 8.4 ^aB^
Arg 4 mM	T_A_	8.9 ± 0.8 ^bB^	84.6 ± 4.4 ^bB^	43.2 ± 4.7 ^cB^	28.4 ± 1.9 ^aC^	165.2 ± 11.6 ^cC^
	T_H_	15.4 ± 1.3 ^aA^	108.0 ± 7.3 ^aA^	55.1 ± 5.9 ^bA^	33.2 ± 3.4 ^aA^	211.6 ± 17.5 ^bA^
	T_E_	17.7 ± 2.7 ^aA^	122.8 ± 5.3 ^aA^	77.0 ± 4.2 ^aA^	22.8 ± 2.8 ^bA^	240.2 ± 14.7 ^aA^
**Main effects**						
Temperature (T)		***	***	***	***	***
Arginine (Arg)		***	***	***	**	***
T × Arg		***	***	***	***	***

Different small letters within a column indicate significant differences between temperatures, and different capital letters within a column, for the same temperature, indicate significant differences between arginine treatments, at *p* = 0.05 (Tukey’s test). Analysis of variance: ** *p* ≤ 0.005; *** *p* ≤ 0.001. Abbreviations: Arg (arginine), T (temperature).

**Table 6:** Antioxidant activity and total phenolic compounds (TPC) of cauliflower florets affected by short-term heat stress and foliar arginine application.

Treatment	TPC (mg GAE g^−1^ FW)			ABTS^•+^ (µg TE g^−1^ DW)		
	T_A_	T_H_	T_E_	T_A_	T_H_	T_E_
Control	77.5 ± 0.5 ^bB^	82.2 ± 3.0 ^bC^	93.8 ± 5.9 ^aC^	38.2 ± 5.6 ^cB^	65.6 ± 1.3 ^bC^	101.0 ± 1.2 ^aB^
Arg 1 mM	83.0 ± 2.9 ^cA^	92.6 ± 1.1 ^bB^	113.2 ± 1.6 ^aB^	39.5 ± 1.3 ^cB^	98.6 ± 14.7 ^bB^	133.6 ± 2.7 ^aA^
Arg 4 mM	84.4 ± 2.1 ^bA^	99.1 ± 13.8 ^bA^	132.4 ± 9.6 ^aA^	84.7 ± 10.4 ^cA^	113.6 ± 12.2 ^bA^	138.7 ± 5.9 ^aA^
**Main effects**						
Temperature (T)	***			***		
Arginine (Arg)	***			***		
T × Arg	**			***		

Different small letters within a row indicate significant differences between temperatures, and different capital letters within a column, for the same temperature, indicate significant differences between arginine treatments, at *p* = 0.05 (Tukey’s test). Analysis of variance: ** *p* ≤ 0.005; *** *p* ≤ 0.001. Abbreviations: Arg (arginine), GAE (gallic acid equivalents), T (temperature), TE (Trolox equivalents).

**Table 7:** Polyamine content (nmoles g^−1^ FW) of cauliflower florets affected by short-term heat stress and foliar arginine application.

Treatment	Temp	Putrescine	Cadaverine	Spermidine	Spermine	Total
Control	T_A_	3.2 ± 0.5 ^cC^	11.2 ± 0.5 ^bA^	1.9 ± 0.3 ^cC^	4.88 ± 0.9 ^cB^	21.1 ± 2 ^cC^
	T_H_	22.6 ± 2.6 ^bC^	13.4 ± 2.6 ^bA^	4.3 ± 0.6 ^bC^	6.6 ± 0.7 ^bA^	46.8 ± 5.9 ^bC^
	T_E_	42.5 ± 3.2 ^aC^	17.4 ± 4.0 ^aC^	7.9 ± 0.6 ^aB^	8.49 ± 0.5 ^aC^	76.3 ± 7.5 ^aC^
Arg 1 mM	T_A_	7.9 ± 0.9 ^cB^	11.4 ± 0.5 ^cA^	5.6 ± 0.6 ^cB^	6.5 ± 0.7 ^bA^	31.4 ± 2.4 ^cB^
	T_H_	34.8 ± 7.2 ^bB^	13.7 ± 2.6 ^bA^	7.8 ± 0.5 ^bB^	6.8 ± 0.9 ^bA^	63.1 ± 10.1 ^bB^
	T_E_	71.0 ± 2.7 ^aA^	23.1 ± 2.7 ^aB^	8.9 ± 0.5 ^aB^	23.3 ± 2.7 ^aB^	126.2 ± 7.7 ^aB^
Arg 4 mM	T_A_	17.1 ± 1.9 ^bA^	12.0 ± 0.5 ^cA^	11.0 ± 1.0 ^cA^	6.0 ± 0.5 ^bA^	46.2 ± 3.5 ^cA^
	T_H_	44.1 ±4.0 ^bA^	15.8 ± 4.0 ^bA^	13.7 ± 1.4 ^bA^	7.8 ± 0.5 ^bA^	80.4 ± 8.9 ^bA^
	T_E_	124.7 ± 11.3 ^aA^	26.7 ± 7.2 ^aA^	80.9 ± 1.7 ^aA^	26.8 ± 2.1 ^aA^	259.1 ± 20.1 ^aA^
**Main effects**						
Temperature (T)		***	***	***	***	***
Arginine (Arg)		***	***	***	***	***
T × Arg		***	***	***	***	***

Different small letters within a row indicate significant differences between temperatures, and different capital letters within a row, for the same temperature, indicate significant differences between arginine treatments, at *p* = 0.05 (Tukey’s test). Analysis of variance: *** *p* ≤ 0.001. Abbreviations: Arg (arginine), T (temperature).
